# Supplementary material for: Identification of core genes in the extracellular matrix and the regulatory mechanisms of the immune microenvironment in idiopathic pulmonary fibrosis using WGCNA and machine learning methods
Source: PLoS One. 2025 Aug 26;20(8):e0330725. doi: 10.1371/journal.pone.0330725 (PMC12380308; doi:10.1371/journal.pone.0330725)
Supplement: Attachment — Submitted filename: Response to Reviewers.docx [file pone.0330725.s002.docx]

Dear Editors and Reviewers:

Thank you for your letter and for the reviewers’ comments concerning our manuscript entitled “Identification of Core Genes in the Extracellular Matrix and the Regulatory Mechanisms of the Immune Microenvironment in Idiopathic Pulmonary Fibrosis Using WGCNA and Machine Learning Methods” (ID: PONE-D-25-20083). Those comments are all valuable and very helpful for revising and improving our paper, as well as having important guiding significance for our research. We have studied the comments carefully and have made corrections which we hope will meet with approval. The revised portions are marked in red in the paper. The main corrections in the paper and our responses to the reviewers’ comments are as follows:

Responses to Editor's and reviewers' comments:

Editor:

1. Response to comment: The format requirements of PLOS ONE

Response:We have carefully revised the manuscript to comply with the journal’s style guidelines.

2. Response to comment: code

Response:I sincerely apologize for the current limitations in my understanding of the code sharing requirements, and I have not yet been able to obtain access to the shared code. As a result, I am temporarily unable to provide the relevant code. However, I have carefully reviewed the code sharing guidelines and would be more than willing to share the code if it becomes available in the future.

3. Response to comment:With regard to the issue of text overlap in previously published works

Response:We have carried out a comprehensive examination of the article's content and ascertained that the major areas of text overlap are concentrated in the description of methods and publicly accessible information related to diseases. During the writing process, we have made citations of the articles mentioned by the editor in strict accordance with academic norms. To decrease the similarity rate, we have rewritten the paragraphs with the highest similarity levels, specifically the first paragraph of the introduction section and the first paragraph of the discussion section.

**Delete:**Idiopathic pulmonary fibrosis (IPF) is a chronic, progressive, and ultimately fatal interstitial lung disease predominantly affecting older adults. It is characterized by worsening dyspnea and a continuous decline in lung function, with high mortality rates that pose significant challenges for clinical management [1]. Although the exact etiology of IPF remains unclear, evidence indicates that genetic predispositions and environmental exposures play critical roles in its pathogenesis. Recent studies have highlighted the extracellular matrix (ECM) as a pivotal factor driving pulmonary remodeling and fibrotic progression [2]. Despite these insights, current therapeutic options remain limited, with no definitive cure available. While medications such as nintedanib and pirfenidone can slow disease progression, their use is often constrained by adverse effects [3]. Therefore, further investigation into the molecular mechanisms and signaling pathways underlying IPF is essential for advancing treatment strategies.
**Be revised to:**Idiopathic pulmonary fibrosis (IPF) is a fatal interstitial lung disease with a high incidence among the elderly, characterized by progressive respiratory function deterioration and high mortality, presenting significant challenges in clinical management [1]. Research indicates that genetic susceptibility (such as MUC5B promoter polymorphism) and environmental exposure (dust, pollutants) interactively drive the onset of the disease, with extracellular matrix (ECM) remodeling [2] and immune microenvironment dysregulation playing a core role. Although existing drugs (nintedanib/pirfenidone) can slow down the progression [3], their efficacy is limited and side effects are significant, making it urgent to achieve breakthroughs in targeted therapy. Therefore, in-depth exploration of the molecular mechanisms and signaling pathways of IPF is crucial for advancing therapeutic strategies.
**Delete:**When the lung sustains injury, the fibrosis process begins and the disease continues to progress. IPF is a lethal, progressive fibrosing parenchymal lung disease that affects millions of patients worldwide and is refractory to most treatment options. Fibrosis may play a critical role in the later stages of disease progression, which could be the result of the interaction of multiple pathogenic factors, with ECM[30] remodeling and immune cell infiltration playing crucial roles. Non-invasive genetic analysis not only provides critical diagnostic information but is also essential for understanding the progression of IPF.

**Be revised to:**Idiopathic pulmonary fibrosis (IPF) is a fatal interstitial lung disease with a high incidence among the elderly, characterized by progressive respiratory function deterioration and high mortality, presenting significant challenges in clinical management [1]. Research indicates that genetic susceptibility (such as MUC5B promoter polymorphism) and environmental exposure (dust, pollutants) interactively drive the onset of the disease, with extracellular matrix (ECM) remodeling [2] and immune microenvironment dysregulation playing a core role. Although existing drugs (nintedanib/pirfenidone) can slow down the progression [3], their efficacy is limited and side effects are significant, making it urgent to achieve breakthroughs in targeted therapy. Therefore, in-depth exploration of the molecular mechanisms and signaling pathways of IPF is crucial for advancing therapeutic strategies.

4.Response to comment:ORCID iD

Response：The ORCID of the corresponding author has now been duly updated.

5.Response to comment:An error in the funding information

Response：We sincerely appreciate your reminder. The funding information within the manuscript is accurate; however, an error transpired during the submission procedure. We will promptly rectify this when we resubmit the manuscript.

6.Response to comment:The author encountered an oversight during the submission process.

Response：We will duly rectify the issue upon resubmission.

7.Response to comment:The problem of picture clarity

Response：All figures have been re-edited in strict compliance with the journal's requirements. Moreover, their clarity has been appropriately adjusted, and they have been re-uploaded accordingly.

8.Response to comment:Figure 13 is not cited in the main text.

Response：We sincerely regret any inconvenience caused by our failure to thoroughly verify the chart information prior to submission. We have conducted a comprehensive review of all chart attributes, image clarity, and in-text citations, and have implemented the necessary corrections in the revised manuscript. With regard to Figure 13, we confirm that it is appropriately included in the main text and has been correctly cited.

9.Response to comment:Figure Upload Requirements

Response：Figures have been uploaded separately as per submission guidelines.

10.Response to comment:Supporting Information files

Response：We will comply with the upload requirements as specified.

11.Response to comment:The references suggested by the reviewers

Response：We have meticulously evaluated the references recommended by the reviewers. We confirm that they are highly congruent with the direction of our research and hold substantial reference value. Appropriate citations have been incorporated into the revised manuscript.

Reviewer #1:

Major Comments：Employ scRNA-seq technology to identify the cellular origins of key genes.

Response:We sincerely appreciate the insightful feedback provided. This study was conducted based on bioinformatics approaches. The initial exploration of key genes in the manuscript was relatively limited. However, your comments offered crucial theoretical insights into the cellular origins of BAAT, COMP, and CXCL13. We have thoroughly reviewed the relevant literature and confirmed that the expression patterns of these three genes align closely with existing single-cell RNA sequencing (scRNA-seq) data. In response to your suggestions, we have substantially revised the Discussion section to provide a comprehensive analysis of each gene’s cellular origin, potential molecular mechanisms, and their associations with immune processes.

**Delete:**Following single-gene analyses of *BAAT*, *COMP*, and *CXCL13*, we observed that the expression levels of all three genes were significantly elevated in the IPF group, demonstrating their potential as robust diagnostic markers. At the molecular level, *COMP,* a key regulator of ECM remodeling, has been mechanistically linked to pro-fibrotic processes in macrophage-fibroblast interactions, as demonstrated by Vuga LJ et al. *COMP* is a non-collagenous ECM protein and one of the most significantly upregulated genes in IPF lungs. This finding was corroborated at the protein level through Western blot analysis. Immunohistochemical staining revealed that *COMP* is expressed in dense fibrotic regions of IPF lungs, co-localizing with vimentin and surrounding cells expressing *pSMAD3*. Compared to controls, ELISA measurements showed a significant increase in *COMP* protein concentration in the serum of IPF patients. The chemokine *CXCL13* plays a critical role in the formation of tertiary lymphoid structures (TLS). Zhao T et al. [36] recently confirmed its involvement in generating ectopic lymphoid follicles in the lungs via the *CXCR5/BCA-1* axis. We will employ multiplex immunofluorescence to map its spatial expression in idiopathic pulmonary fibrosis lung tissue. Notably, *BAAT* emerged as a novel candidate gene in our study. Its potential link to lung ECM remodeling through bile acid metabolism remains unclear. Although Chen et al. [37] found that bile acids induce the activation of alveolar epithelial cells and lung fibroblasts through Farnesoid X receptor-dependent and -independent pathways, thereby contributing to pulmonary fibrosis, the regulatory role of *BAAT* in *MMP* activity or collagen cross-linking in IPF requires further investigation through metabolomics-transcriptomics analysis, offering a promising direction for future research.

**Be revised to:**Following single-gene analyses of *BAAT*, *COMP*, and *CXCL13*, we observed that the expression levels of all three genes were significantly elevated in the IPF group, demonstrating their potential as robust diagnostic markers. The bile acid-CoA:amino acid N-acyltransferase, encoded by the *BAAT* gene, catalyzes the conjugation of primary bile acids—such as cholic acid and chenodeoxycholic acid—with glycine or taurine via amide bonds in the liver, resulting in the formation of conjugated bile acids. This biochemical process represents a key step in bile acid metabolism. Historically, the liver has been considered the primary site of *BAAT* activity, with bile acids detected in lung tissue believed to originate either from hepatic synthesis followed by systemic circulation or to be indirectly regulated by gut microbiota through the gut-lung axis. However, accumulating evidence suggests that under pathological conditions, *BAAT* expression and its potential functions in the lungs have increasingly attracted scientific attention.Chen et al. [37] reported activation of the bile acid signaling pathway in the lung tissue of individuals with pulmonary fibrosis, primarily mediated through the FXR receptor, suggesting the possibility of localized bile acid metabolic disturbances. This hypothesis is further supported by single-cell transcriptomic data. Public databases, such as the Human Protein Atlas, indicate that *BAAT* mRNA expression is minimal in healthy lung tissue but significantly elevated in patients with IPF. Adams TS et al. [38] further demonstrated that *BAAT* expression in IPF lungs exhibits distinct cellular specificity, with transcriptional signals predominantly localized in aberrant basal-like cell populations. Histopathological analysis reveals that these cells are specifically located at the leading edges of myofibroblast foci and display unique molecular features, including co-expression of epithelial markers (KRT5 and TP63), basal cell markers (NGFR and ITGA6), epithelial-mesenchymal transition (EMT)-related genes (VIM and CDH2), and senescence-associated genes (CDKN2A). Notably, these cells also exhibit high expression of the transcription factor SOX9, which has been identified as a key regulator of distal airway development and tissue repair.Moreover, Habermann AC et al. [39] observed that *BAAT* is specifically enriched in KRT5−/KRT17+ cells—a pathological cell population associated with excessive ECM production. This phenotype is driven by activation of the SOX4/SOX9 pathway and suppression of the NR1D1 signaling network. RNA in situ hybridization (RNA-ISH) results further confirm significant co-localization of KRT17 and SOX9 in fibrotic lung tissues, providing direct evidence for the involvement of dysregulated epithelial cells in collagen synthesis.In summary, *BAAT* is markedly upregulated in the lungs of IPF patients, and its cellular origin is closely associated with pathological airway remodeling and abnormal ECM accumulation. We propose that the enzyme encoded by *BAAT* may exhibit catalytic activity in the lung and contribute to pulmonary fibrosis through several potential mechanisms:1. Local bile acid signaling pathway [37]. Conjugated bile acids generated by *BAAT* may activate alveolar epithelial cells or fibroblasts via FXR or TGR5 receptors, thereby promoting the expression and release of pro-fibrotic mediators such as TGF-β and CTGF.2. Immune-metabolic interaction pathway [40]: Conjugated bile acids may modulate the function of lung immune cells, including Th17 cells and macrophages, thereby indirectly promoting ECM deposition.3. Oxidative stress and apoptosis pathway [41]: Studies have shown that conjugated bile acids accumulate in T cells of liver cancer patients, impairing mitochondrial respiration and inducing reactive oxygen species (ROS) production. We speculate that similar pathophysiological changes may occur in the lungs of IPF patients, where bile acid metabolic imbalance may lead to ROS generation, subsequently activating pro-fibrotic enzymes such as lysyl oxidase (LOX), exacerbating collagen cross-linking (in synergy with MMP/TIMP imbalance), and inducing apoptosis.Despite these insights, significant gaps remain in understanding the precise role of BAAT in IPF. First, the conjugation of bile acids requires the activation of primary bile acids and coenzyme A (CoA), yet the lung lacks the complete bile acid synthesis enzyme system (e.g., CYP7A1, CYP27A1). Whether the lung can sustain local bile acid metabolism through the uptake of circulating primary bile acids remains to be determined. Second, although *BAAT* is expressed in the lung, its baseline levels are very low, raising doubts about whether the resulting conjugated bile acids are sufficient to exert meaningful physiological effects.Therefore, future studies should focus on clarifying the functional status of *BAAT* in lung tissue: examining *BAAT* protein expression via immunohistochemistry or Western blotting; evaluating its enzymatic activity using lung tissue homogenates; and comparing the ratios of conjugated to free bile acids in IPF and control lung tissues using metabolomic approaches. Given the low concentrations of bile acids in lung tissue, such analyses will likely require highly sensitive techniques, such as liquid chromatography-mass spectrometry (LC-MS).In conclusion, although the upregulation of *BAAT* in the lungs of IPF patients has been documented, there is currently no direct evidence confirming its catalytic activity in this organ. If such activity is validated, it could open new avenues for understanding the metabolic-immune regulatory mechanisms in IPF, offering both theoretical and clinical implications.

The *COMP* gene encodes cartilage oligomeric matrix protein (COMP), a non-collagenous glycoprotein belonging to the thrombospondin family and functioning as a key component of ECM. Single-cell RNA sequencing (scRNA-seq) data demonstrate that *COMP* is predominantly expressed in myofibroblasts and fibroblasts and represents one of the most significantly upregulated genes in lung tissues from patients with IPF. Compared to healthy controls, COMP expression levels are reported to increase by approximately 17-fold [38], a result that has been validated at the protein level using Western blot analysis. Immunohistochemical staining further reveals that *COMP* is highly localized in the dense fibrotic regions of IPF lungs, where it co-localizes with vimentin and is spatially associated with cells expressing phosphorylated SMAD3 (pSMAD3). ELISA measurements also show that serum COMP levels are significantly elevated in IPF patients compared to healthy individuals.From a pathogenic perspective, IPF is characterized by excessive fibroblast proliferation, enhanced differentiation into myofibroblasts, and excessive deposition of collagen. *COMP*’s marked overexpression in these cell types underscores its critical involvement in the fibrotic process at the cellular level. These observations are consistent with findings from studies by Adams TS et al. [38] and Habermann AC et al. [39]. Further mechanistic insights were provided by Vuga LJ et al. [42], who demonstrated that *COMP* promotes fibroblast activation through upregulation of TGF-β1 and enhanced SMAD3 phosphorylation *(pSMAD3*↑), thereby facilitating their transformation into myofibroblasts. This leads to increased expression of *α-SMA* and *COL1A1* and suppression of MMP activity, ultimately reducing ECM degradation. Experimental knockdown of *COMP* has been shown to significantly inhibit the expression of these fibrotic markers in pulmonary fibroblasts. Moreover, *COMP* plays a central role in mediating pro-fibrotic crosstalk between macrophages and fibroblasts.

*CXCL13* is a member of the CXC chemokine family, primarily secreted by stromal cells in lymphoid tissues. It specifically binds to the CXCR5 receptor, guiding the migration of B cells and follicular helper T cells (Tfh) into lymphoid follicles [43]. In the context of IPF, *CXCL13* promotes the aggregation of B and T cells within lung tissue, contributing to the formation of ectopic lymphoid follicles. These structures are implicated in antigen presentation, autoantibody production, and the organization of TLS [44]. Research by Nessrine Bellamri et al. [45] identified CD68+ and CD206+ alveolar macrophages as the primary cellular source of *CXCL13* in IPF patients. This finding was further supported by Morse et al. [46], who used single-cell RNA sequencing to show that *CXCL13* is significantly upregulated in abnormal basal cell populations, providing strong evidence for its cellular origin.Asai Y et al. [47] demonstrated that a subset of CD4+ T cells, specifically Tfh cells, expresses the CXCR5 receptor. Upon binding to *CXCL13*, this interaction facilitates the migration of Tfh cells into B cell follicles. These Tfh cells express co-stimulatory molecules such as ICOS and immune regulatory molecules like PD-1, and utilize BCL6 as a master transcription factor to secrete cytokines including IL-4, IL-10, and IL-21, which are essential for B cell activation, differentiation, and class switching. Excessive activation of Tfh cells may lead to antigen-specific antibody responses and potentially contribute to autoimmune phenomena. Notably, Morse et al. [46] also observed significant alterations in the CD4+ Treg subset (FOXP3+) in IPF lung tissues compared to controls, suggesting a potential role for regulatory T cells in disease pathogenesis.In addition to its immunomodulatory functions, CXCL13 can activate lung fibroblasts via the CXCR5 receptor, leading to upregulation of α-SMA and type I collagen expression and thereby promoting extracellular matrix accumulation. CXCL13 also synergizes with TGF-β to enhance its pro-fibrotic effects through increased SMAD3 phosphorylation. Recent clinical studies have shown that serum CXCL13 levels are significantly elevated in IPF patients and are positively correlated with disease severity and progression rate [48].

Minor Comments

1. Response to comment: Figure 9A (Immune Cell Infiltration)

Response: We sincerely express our gratitude for your invaluable comments. Regarding the original Figure 9A, there was an issue of incomplete annotation in the analysis results of immune cell infiltration. Similar to Figure 9B, this figure presented comparative data between the IPF group and the control group. However, during the visualization process, the group information was not clearly labeled.We have systematically revised the relevant figures. In the revised Figure 9, we have added independent labels and comparative annotations for the IPF group and the control group. This ensures that readers can accurately distinguish the differences in cell infiltration characteristics between the experimental and control groups, thereby significantly enhancing the interpretability of the data.

2. Response to comment:Clarity and Readability of Figures and Legends:

Response:We sincerely appreciate your identification of these critical issues. Upon careful inspection, we confirm that during the format conversion of the manuscript, technical glitches occurred, specifically a degradation in image resolution and the absence of certain identifiers.We have carried out a comprehensive and systematic revision of all figures and tables. This involved enhancing the image resolution, adding essential identifiers, and optimizing the visual presentation through professional layout techniques.Regarding the concerns you raised about the imprecise and redundant legend information, we have addressed this meticulously. The legend of Figure 7 has been fully revised in accordance with your suggestions. It should be noted that, to maintain the integrity of the methodological description, the legend of Figure 2 remains unchanged. For the rest of the figures, we have implemented standardized revisions to ensure that the legend information adheres to the journal's stringent criteria of conciseness and accuracy.

Please refer to the revised draft for specific changes.

3.Response to comment:Clarification of Datasets in Methods:

Response：In pursuit of greater precision in the text, we have implemented modifications in accordance with your valuable insights.

**Delete:**Eventually, GSE150910 and GSE70866 were identified and collected for further analysis.

**Be revised to:**Ultimately, two datasets derived from lung tissue samples of patients with idiopathic pulmonary fibrosis (IPF) and healthy control individuals were screened and acquired: GSE150910 (an RNA-seq dataset) and GSE70866 (a microarray dataset). Subsequently, further analyses were performed on these datasets

4.Response to comment:Wording of Correlation Analysis Results

Response：We sincerely express our gratitude for your meticulous and rigorous reviewing comments. It is undeniable that there were inaccuracies in the presentation of this part. In light of your suggestions, we have made targeted modifications to enhance the clarity and scientific soundness of the discourse.

**Delete:**immune cells with higher expression

**Be revised to:**immune cell composition in samples with elevated gene expression

We hereby extend our heartfelt gratitude for your thorough and incisive peer review of this research. Your profound insights into Idiopathic Pulmonary Fibrosis (IPF) and meticulous validation of every technical element within the research design have substantially elevated the academic rigor and theoretical profundity of this study.Specifically, your stringent scrutiny of the rationality and validity of IPF bioinformatics analysis, coupled with the constructive recommendations regarding the cytological evidence of key genes, have effectively enhanced the internal consistency of the research logic and the integrity of the argumentative framework.Under your expert guidance, the theoretical applicability of this research in elucidating the pathogenesis of IPF has been remarkably fortified, thereby contributing significantly to the advancement of knowledge in this field.Special thanks to you for your good comments.

Reviewer #2:

1. Response to comment:The Importance of Investigating Key Genes and the Anticipated Outcomes

Response：We hereby express our sincere gratitude for your peer review comments, which are of significant academic value. Through systematic literature reviews and multiple validation analyses, we have restructured this section of the content, presenting it from the following two dimensions:First, in terms of single-gene traceability analysis using single-cell sequencing technology, we have now supplemented cell-specific expression profiles and spatial localization validation data (see paragraphs 5, 6, and 7 in the Discussion section of the revised manuscript).Second, at the end of the article, we have incorporated a section highlighting the importance and expected outcomes of this multi-dimensional bioinformatics analysis in understanding Idiopathic Pulmonary Fibrosis (IPF) (see paragraph 4 in the Deficiencies and Further Perspectives section of the revised manuscript).

2. Response to comment:The cellular source of CXCL13

Response：References and explanations are provided in Paragraph 7 of the Discussion section.

Add text:*CXCL13* is a member of the CXC chemokine family, primarily secreted by stromal cells in lymphoid tissues. It specifically binds to the CXCR5 receptor, guiding the migration of B cells and follicular helper T cells (Tfh) into lymphoid follicles [43]. In the context of IPF, *CXCL13* promotes the aggregation of B and T cells within lung tissue, contributing to the formation of ectopic lymphoid follicles. These structures are implicated in antigen presentation, autoantibody production, and the organization of TLS [44]. Research by Nessrine Bellamri et al. [45] identified CD68+ and CD206+ alveolar macrophages as the primary cellular source of *CXCL13* in IPF patients. This finding was further supported by Morse et al. [46], who used single-cell RNA sequencing to show that *CXCL13* is significantly upregulated in abnormal basal cell populations, providing strong evidence for its cellular origin.Asai Y et al. [47] demonstrated that a subset of CD4+ T cells, specifically Tfh cells, expresses the CXCR5 receptor. Upon binding to *CXCL13*, this interaction facilitates the migration of Tfh cells into B cell follicles. These Tfh cells express co-stimulatory molecules such as ICOS and immune regulatory molecules like PD-1, and utilize BCL6 as a master transcription factor to secrete cytokines including IL-4, IL-10, and IL-21, which are essential for B cell activation, differentiation, and class switching. Excessive activation of Tfh cells may lead to antigen-specific antibody responses and potentially contribute to autoimmune phenomena. Notably, Morse et al. [46] also observed significant alterations in the CD4+ Treg subset (FOXP3+) in IPF lung tissues compared to controls, suggesting a potential role for regulatory T cells in disease pathogenesis.In addition to its immunomodulatory functions, CXCL13 can activate lung fibroblasts via the CXCR5 receptor, leading to upregulation of α-SMA and type I collagen expression and thereby promoting extracellular matrix accumulation. CXCL13 also synergizes with TGF-β to enhance its pro-fibrotic effects through increased SMAD3 phosphorylation. Recent clinical studies have shown that serum CXCL13 levels are significantly elevated in IPF patients and are positively correlated with disease severity and progression rate [48].

3. Response to comment:The mechanism of action of BAAT

Response：We sincerely appreciate your insightful review comments. In response to your inquiries, we conducted a comprehensive review of relevant literature and identified preliminary theoretical foundations. Based on the known mechanisms of action in liver diseases, we have proposed a plausible hypothesis. Furthermore, we have clearly outlined the current limitations of this hypothesis and highlighted key questions that warrant further investigation in future studies (please refer to Paragraph 5 of the Discussion section for details).

Add text:The bile acid-CoA:amino acid N-acyltransferase, encoded by the *BAAT* gene, catalyzes the conjugation of primary bile acids—such as cholic acid and chenodeoxycholic acid—with glycine or taurine via amide bonds in the liver, resulting in the formation of conjugated bile acids. This biochemical process represents a key step in bile acid metabolism. Historically, the liver has been considered the primary site of *BAAT* activity, with bile acids detected in lung tissue believed to originate either from hepatic synthesis followed by systemic circulation or to be indirectly regulated by gut microbiota through the gut-lung axis. However, accumulating evidence suggests that under pathological conditions, *BAAT* expression and its potential functions in the lungs have increasingly attracted scientific attention.Chen et al. [37] reported activation of the bile acid signaling pathway in the lung tissue of individuals with pulmonary fibrosis, primarily mediated through the FXR receptor, suggesting the possibility of localized bile acid metabolic disturbances. This hypothesis is further supported by single-cell transcriptomic data. Public databases, such as the Human Protein Atlas, indicate that *BAAT* mRNA expression is minimal in healthy lung tissue but significantly elevated in patients with IPF. Adams TS et al. [38] further demonstrated that *BAAT* expression in IPF lungs exhibits distinct cellular specificity, with transcriptional signals predominantly localized in aberrant basal-like cell populations. Histopathological analysis reveals that these cells are specifically located at the leading edges of myofibroblast foci and display unique molecular features, including co-expression of epithelial markers (KRT5 and TP63), basal cell markers (NGFR and ITGA6), epithelial-mesenchymal transition (EMT)-related genes (VIM and CDH2), and senescence-associated genes (CDKN2A). Notably, these cells also exhibit high expression of the transcription factor SOX9, which has been identified as a key regulator of distal airway development and tissue repair.Moreover, Habermann AC et al. [39] observed that *BAAT* is specifically enriched in KRT5−/KRT17+ cells—a pathological cell population associated with excessive ECM production. This phenotype is driven by activation of the SOX4/SOX9 pathway and suppression of the NR1D1 signaling network. RNA in situ hybridization (RNA-ISH) results further confirm significant co-localization of KRT17 and SOX9 in fibrotic lung tissues, providing direct evidence for the involvement of dysregulated epithelial cells in collagen synthesis.In summary, *BAAT* is markedly upregulated in the lungs of IPF patients, and its cellular origin is closely associated with pathological airway remodeling and abnormal ECM accumulation. We propose that the enzyme encoded by *BAAT* may exhibit catalytic activity in the lung and contribute to pulmonary fibrosis through several potential mechanisms:1. Local bile acid signaling pathway [37]. Conjugated bile acids generated by *BAAT* may activate alveolar epithelial cells or fibroblasts via FXR or TGR5 receptors, thereby promoting the expression and release of pro-fibrotic mediators such as TGF-β and CTGF.2. Immune-metabolic interaction pathway [40]: Conjugated bile acids may modulate the function of lung immune cells, including Th17 cells and macrophages, thereby indirectly promoting ECM deposition.3. Oxidative stress and apoptosis pathway [41]: Studies have shown that conjugated bile acids accumulate in T cells of liver cancer patients, impairing mitochondrial respiration and inducing reactive oxygen species (ROS) production. We speculate that similar pathophysiological changes may occur in the lungs of IPF patients, where bile acid metabolic imbalance may lead to ROS generation, subsequently activating pro-fibrotic enzymes such as lysyl oxidase (LOX), exacerbating collagen cross-linking (in synergy with MMP/TIMP imbalance), and inducing apoptosis.Despite these insights, significant gaps remain in understanding the precise role of BAAT in IPF. First, the conjugation of bile acids requires the activation of primary bile acids and coenzyme A (CoA), yet the lung lacks the complete bile acid synthesis enzyme system (e.g., CYP7A1, CYP27A1). Whether the lung can sustain local bile acid metabolism through the uptake of circulating primary bile acids remains to be determined. Second, although *BAAT* is expressed in the lung, its baseline levels are very low, raising doubts about whether the resulting conjugated bile acids are sufficient to exert meaningful physiological effects.Therefore, future studies should focus on clarifying the functional status of *BAAT* in lung tissue: examining *BAAT* protein expression via immunohistochemistry or Western blotting; evaluating its enzymatic activity using lung tissue homogenates; and comparing the ratios of conjugated to free bile acids in IPF and control lung tissues using metabolomic approaches. Given the low concentrations of bile acids in lung tissue, such analyses will likely require highly sensitive techniques, such as liquid chromatography-mass spectrometry (LC-MS).In conclusion, although the upregulation of *BAAT* in the lungs of IPF patients has been documented, there is currently no direct evidence confirming its catalytic activity in this organ. If such activity is validated, it could open new avenues for understanding the metabolic-immune regulatory mechanisms in IPF, offering both theoretical and clinical implications.

We sincerely appreciate your rigorous peer review of this research. Your in-depth evaluation of methodological validity and constructive feedback on the functional characterization of molecular targets have substantially strengthened both the theoretical framework and empirical robustness of this study. In response to the key issues you raised, we conducted a comprehensive review of relevant literature, enriched the theoretical foundation, and performed an in-depth, systematic investigation into the pathological mechanisms of idiopathic pulmonary fibrosis (IPF). Your comprehensive and systematic perspectives on bioinformatics methodologies have provided valuable guidance for our future research directions. We are deeply grateful for your professional and insightful review comments.

Other changes:

1. The title page has been reformatted in accordance with the journal's submission guidelines.

2. The fifth paragraph of the Introduction contains numerous repetitive expressions. These have been rewritten to enhance the conciseness and logical coherence of the expression.

**Delete:**Recent research emphasizes the central role of the extracellular matrix (ECM) in mediating pulmonary remodeling and fibrosis. IPF pathogenesis involves the interaction of genetic and environmental factors. Given the limited treatment options and incurability of IPF, early and accurate diagnosis is critical for effective disease management. This study focuses on discovering and clinically applying novel biomarkers for early IPF detection. Integrating biomarkers with clinical parameters allows monitoring disease progression and evaluating treatment responses, enabling personalized strategies for timely intervention to slow or halt disease progression.

**Be revised to:This study aims to screen IPF-specific biomarkers through bioinformatics, and construct a multi-parameter monitoring system to guide early diagnosis and personalized treatment. Integrating biomarkers with clinical data can precisely assess disease stages, providing a theoretical basis for the development of novel therapies targeting ECM remodeling and signal pathway regulation.**

3. All figure captions have been updated from "FIGURE" to "Fig" to ensure consistency with standard academic formatting conventions.

4.As a result of revising the discussion section, ten new references have been inserted starting from the 37th entry, and all subsequent reference numbers have been adjusted accordingly.

We have made every effort to improve the manuscript by implementing certain revisions, which do not affect the overall content or structure of the paper. These changes have been highlighted in red in the revised version and are not listed here.

We sincerely appreciate the Editors’ and Reviewers’ dedicated and thoughtful work. We hope the revised manuscript meets your approval.

Once again, thank you very much for your valuable comments and suggestions.

Best regards,

Shihuan Yu
